# Supplementary material for: CaGβ Promotes CaWRKY40 to Activate Immunity Against Ralstonia solanacearum but Disables It from Activating Thermotolerance
Source: Plants (Basel). 2025 Dec 29;15(1):101. doi: 10.3390/plants15010101 (PMC12787344; doi:10.3390/plants15010101)
Supplement: Supplementary file 1 [file plants-15-00101-s001.zip › plants-4027371-supplementary.pdf]

Table S1 Vector construction and fluorescent quantitative Primer Sequences

name	Primer Sequences
CaGβ-F	GGGGACAAGTTTGTACAAAAAAGCAGGCTTC<br>ATGGCGCAAGAATCACTTGT
CaGβ-R	GGGGACCACTTTGTACAAGAAAGCTGGGTC<br>CTAATAACGACCAATACCCC
CaGβ-T-R	GGGGACCACTTTGTACAAGAAAGCTGGGTC<br>ATAACGACCAATACCCC
CaGβ-V-F	GGGGACAAGTTTGTACAAAAAAGCAGGCTTC<br>ATGGCGCAAGAATCACTTGT
CaGβ-V-R	GGGGACCACTTTGTACAAGAAAGCTGGGTC<br>ACGAGCGGTGGTCCCCGCCT
CaGβ-RT-F	GGGTTCATGGGACCGTACTG
CaGβ-RT-R	AACCGCCACAGTGTTGACAT
CaWRKY40-RT-F	CAGGACCAACCATTACTCTCGA
CaWRKY40-RT-R	ATGCTATTGCCACCGGTAGG
CaActin-F	AGGGATGGGTCAAAAGGATGC
CaActin-R	GAGACAACACCGCCTGAATAGC
CaPR1-RT-F	GCCGTGAAGATGTGGGTCAATGA
CaPR1-RT-R	TGAGTTACGCCAGACTACCTGAGTA
CaDEF1-RT-F	GTGAGGAAGAAGTTTGAAAGAAAGTAC
CaDEF1-RT-R	TGCACAGCACTATCATTGCATACAATTC
CaSTH2-RT-F	GTGAGGAAGAAGTTTGAAAGAAAGTAC
CaSTH2-RT-R	TGCACAGCACTATCATTGCATACAATTC
CaNPR1-RT-F	ACTTCTTCGCCGACGCCAAG
CaNPR1-RT-R	GCCAACACATTCACCAGAGCATC

Promoter sequence of ***CaGβ***:

TAATATTTCATTTAGCAAAGTTGTCTTCTCATAGTATTTTCACAGCATATCTAACTTAGTT
GTAATCCATACTATTACCATAGAATTACAATTTAGGCGTGACTTATGCATGCGTATATTT
TCTATGATTTCTATCAGTGTCCGCGGGTTGGTTGCACTGTGTAACAATTTGAGTGTGTTC
AGGTAATCTTTTATATTGTTTAGCTATATGTATAGTTGGACTTCTATTTTCTGGATGGATT
TTCAGAAAGTGAAGGAGACTTTGGCAAGCGATTACAAGGTGAGTAATTGATTCTTTATC
GATTAGGTGGATGTATTGTTGGCAATCAATTGGATCCCTACACTACACTAGGTATGTTG
TATTAAAATATTGTATAACTACATGAATTTCAAGTGGCAGTGTGAATCTTTTGACAAAAA
ATTGTTATCATTCGTGGGTTTATGCTTACATGTCCATCATTGCCTCATTCAACTAACGGG
AAAAGACATCGTTCCACCTCAAACTATATCCGAAAAGTCGAAGCCACACCTAAATTATA
CTAGTGACTTATTACACACCTAAACTATAAAAAAGTGAAACTATTTACACCCTGTCAGG
CCACCACCACTTGCATGTGGTGTAGTATTTTACACGCGCTGCCACGTCAGCACCACGTC
AGTAAAAAGTATCACTTTTTTATAGTTAAGGTGTGTAATAGGTCACTTATATAGTTTAGG
TGTGGATTCGACTTTTCGACTATAGTTTGGGGTGGAAACGATGTCTTTTCCCTTAATGTT

TTTAGCCGTTTACTTTTGTTATTTAATAGGCTGGACGCGCCTAAAATAAGTGTAACACAC
GCGCCTTAGAATGAGGGTCGCGGGAGGTAATAATATCACTTTTATATAGTTAAGGTGTG
TAATAGGTCACTTATATAATTTATGTGTGGCAGAGACTTTTCTTGTATAGTATGGGGTGG
AAATGATGTCTTTTCCCTTCAAATAACTGAAGGTTGATTATGTCACCACGCTTTACATTC
AGGTGTCGGTTGTGTCAGTTCTAGCTTTACATATCTTAAATTCAAATACGGTTAACCATA
TGAATCCTCACTAAACATGAATTCTTAAATTCATGATGTGTGACTTATCATTTACCTAAT
ACTCTTTTGAATACAATATAAGGTATTTTTATGCGTAAATCACTTTCTCACGCATGACCG
TGTAAAAGGATTTATAAGATCTCTATTTATACGTAATTAGTTTTTAGTGACACCTAAAAC
TCCTCGATTCTTTTAACATGTATTACAAGTCAAAGCCCTATATACACATTTAACATTCAA
AACGGATATTGCCCAGTCAGGCCCATTAACATGATCCAATTCCCTTAATGGACCAATAA
CCACAAAACCCTAAAATCAACACACCCTATAAATTCACACTTCCCACAGTTTTAGGTTTT
CTCTTCAGTACCATACAGATCGTTTGTGTCAATTTTGCCTAATCGCCCACCACCTGTTTG
CAAAAA
